# F-box protein Fbxl18 mediates polyubiquitylation and proteasomal degradation of the pro-apoptotic SCF subunit Fbxl7

**DOI:** 10.1038/cddis.2014.585

**Published:** 2015-02-05

**Authors:** Y Liu, T Lear, Y Zhao, J Zhao, C Zou, B B Chen, R K Mallampalli

**Affiliations:** 1 Department of Medicine, the Acute Lung Injury Center of Excellence, University of Pittsburgh, Pittsburgh, PA, USA; 2 Medical Specialty Service Line, Veterans Affairs Pittsburgh Healthcare System, Pittsburgh, PA, USA

## Abstract

Fbxl7, a subunit of the SCF (Skp-Cul1-F-box protein) complex induces mitotic arrest in cells; however, molecular factors that control its cellular abundance remain largely unknown. Here, we identified that an orphan F-box protein, Fbxl18, targets Fbxl7 for its polyubiquitylation and proteasomal degradation. Lys 109 within Fbxl7 is an essential acceptor site for ubiquitin conjugation by Fbxl18. An FQ motif within Fbxl7 serves as a molecular recognition site for Fbxl18 interaction. Ectopically expressed Fbxl7 induces apoptosis in Hela cells, an effect profoundly accentuated after cellular depletion of Fbxl18 protein or expression of Fbxl7 plasmids encoding mutations at either Lys 109 or within the FQ motif. Ectopic expression of Fbxl18 plasmid-limited apoptosis caused by overexpressed Fbxl7 plasmid. Thus, Fbxl18 regulates apoptosis by mediating ubiquitin-dependent proteasomal degradation of the pro-apoptotic protein Fbxl7 that may impact cellular processes involved in cell cycle progression.

It is estimated >80% proteins undergo ubiquitin-dependent degradation either by the proteasome or lysosome.^1^ The ubiquitylation of a target protein occurs in a carefully orchestrated manner by an enzymatic cascade involving an E1 ubiquitin-activating enzyme, an E2-conjugating enzyme and an E3-ubiquitin ligase.^2, 3^ Among hundreds of E3 ligases, the SCF superfamily has emerged as a class of E3 proteins that modulates diverse biological processes.^4^ The SCF complex contains a catalytic core including Skp1, Cul1 and an F-box protein.^5, 6, 7^ The carboxyl terminus of Cul1 recruits the small RING protein Rbx1, which bridges the E2 enzyme to the E3 ligase, and the amino terminus of Cul1 binds to Skp1 and a variable F-box protein as a substrate receptor.^8^ Based on the pivotal roles of SCF E3 ligases in cellular function through the elimination of substrates, SCF components have recently attracted significant attention on cancer therapeutic drug development and potential to modulate inflammation and innate immunity.^9, 12^

F-box proteins contain two domains: an NH_2_-terminal F-box motif and a carboxyl terminal leucine-rich repeat (LRR) motif or tryptophan-aspartic acid (WD) repeat motif, which are identified within two subclasses: Fbxl or Fbxw proteins, respectively^13^ and.^14^ The third subclass of F-box proteins are recognized as Fbxo (F-box only) proteins contain no identifiable LRR or WD motifs. The F-box motif binds to Skp1, whereas the LRR/WD motif is a receptor-like motif that recognizes diverse substrates for ubiquitin ligation and subsequent degradation.^15^ With the increasing characterization of F-box proteins and its cognate substrates, this has led to an improved understanding of how SCF complexes have multiple roles in cell cycle progression, gene transcription, circadian oscillation and inflammation.^16^ However, with the exception of a few SCF components, the substrates for the majority of ~70 F-box proteins remain largely unknown. In addition the mechanisms that regulate the abundance of the majority of F-box proteins remain unclear and this represents an area of active investigation. It is known that some components within the SCF apparatus are targeted by the ubiquitin ligase anaphase-promoting complex or by autoubiquitylation within their own SCF complex.^17, 18^ Recent studies also show that one F-box protein may target another F-box protein for ubiquitylation to mediate protein degradation thereby impairing the SCF activity.^12^

We have previously characterized the role of a relatively new F-box protein, Fbxl7, on cell cycle progression. Fbxl7 within the SCF complex targets Aurora A for polyubiquitylation and proteasomal degradation leading to mitotic cell cycle arrest.^19^ Additionally, Fbxl7 was reported to be associated with an ovarian cancer gene and its expression is linked to steroid responsiveness in human subjects with asthma.^20, 21^ Although these data suggest a role for Fbxl7 in controlling cell proliferative activity and viability, further studies investigating both its biological role and molecular expression are needed. Here we identified that Fbxl7 is itself ubiquitylated and degraded via the proteasome. Interestingly, we identified that an orphan F-box protein, Fbxl18, targets Fbxl7 for its ubiquitylation using distinct molecular signatures that leads to its disposal in cells. Ectopically expressed Fbxl7 displayed pro-apoptotic activity in Hela cells, an effect abrogated by co-expression of Fbxl18. These observations suggest a molecular interplay between two SCF components that govern availability of a biologically important pro-apoptotic protein.

## Results

### Fbxl7 undergoes proteasomal degradation

**Figure 1:**
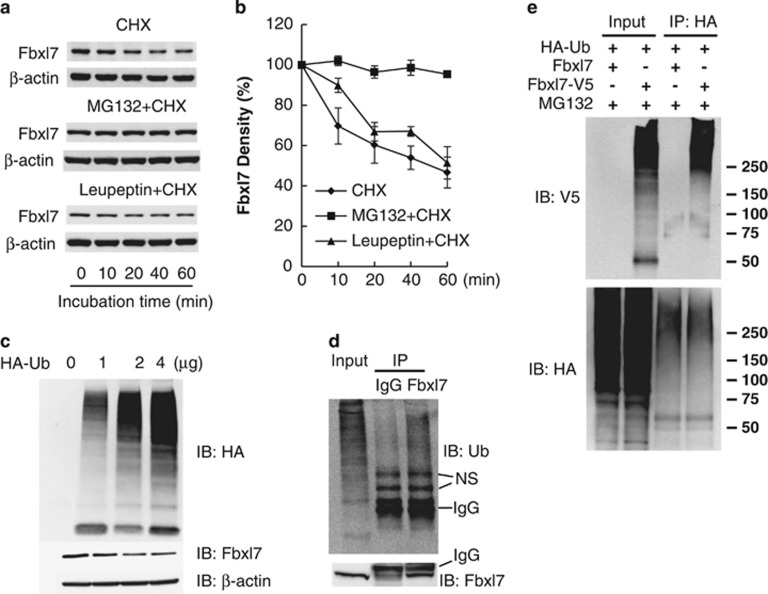
Fbxl7 is polyubiquitylated and undergoes proteasomal degradation.(**a**) MLE12 cells were treated with 20 *μ*g/ml CHX with or without MG-132 (20 *μ*M) or leupeptin (100 *μ*M) for indicated times. Cell lysates were subjected to Fbxl7 and *β*-actin immunoblotting. Each protein band on immunoblots was quantified and shown graphically in (**b**) (data are representative of three independent experiments). (**c**) MLE12 cells were transfected with HA-tagged ubiquitin (0–4 *μ*g), and cells lysates were subjected to HA, Fbxl7 and *β*-actin immunoblotting. (**d**) Co-immunoprecipitation (co-i.p.) studies showing interaction of ubiquitin with Fbxl7. MLE12 cells were treated with MG-132 (20 *μ*M) for 4 h. Cell lysates were subjected to immunoprecipitation (i.p.) with an Fbxl7 antibody. The input cell lysates and the precipitates were probed with ubiquitin antibody and Fbxl7 antibody, respectively. NS: non-specific bands. (**e**) Co-IP studies. MLE12 cells were transfected with plasmids encoding HA-tagged *ubiquitin* (Ub), V5-tagged *Fbxl7*, untagged *Fbxl7*; after 24 h incubation, the cells were exposed to MG-132 (20 *μ*M) for an additional 4 h. Cell lysates were subjected to i.p. with rabbit HA antibody. The input cell lysates and the precipitates were probed with mouse V5 antibody (top) and mouse HA antibody (bottom)

High levels of Fbxl7 in cells appear to critically regulate mitotic arrest; however, the lifespan of this F-box protein in cells is unknown. We first measured the protein stability of endogenous Fbxl7 in MLE12 cells. As shown in Figures 1a and b, cells treated with the protein synthesis inhibitor cycloheximide (CHX) indicated that the half-life of Fbxl7 is ~1 h. To identify the degradation pathway involved in Fbxl7 decay, MLE12 cells were also treated with the proteasome inhibitor (MG132) or a lysosome inhibitor (leupeptin) prior to CHX treatment. MG132 treatment, instead of leupeptin, resulted in accumulation of immunoreactive Fbxl7 in cells, which suggests that the proteasome, rather than the lysosome is involved in degradation of Fbxl7.

### Fbxl7 is polyubiquitylated

To test if Fbxl7 is modified by ubiquitylation, we examined steady-state levels of Fbxl7 protein after overexpression of cells with a plasmid encoding ubiquitin. The levels of endogenous Fbxl7 decreased after expression of increasing amounts of ectopically expressed HA-tagged ubiquitin (Figure 1c). In co-immunoprecipitation (co-i.p.) studies, we also detected an interaction between endogenous Fbxl7 and ubiquitin (Figure 1d). We further confirmed the binding of Fbxl7 to ubiquitin after expression in cells with *Fbxl7*-V5 and HA-*ubiquitin* (HA-Ub) plasmids under MG132 treatment (Figure 1e). Collectively, these results indicate that Fbxl7 degradation is mediated by the ubiquitin-proteasome system.

### Fbxl18 targets Fbxl7 for its ubiquitylation and degradation

**Figure 2:**
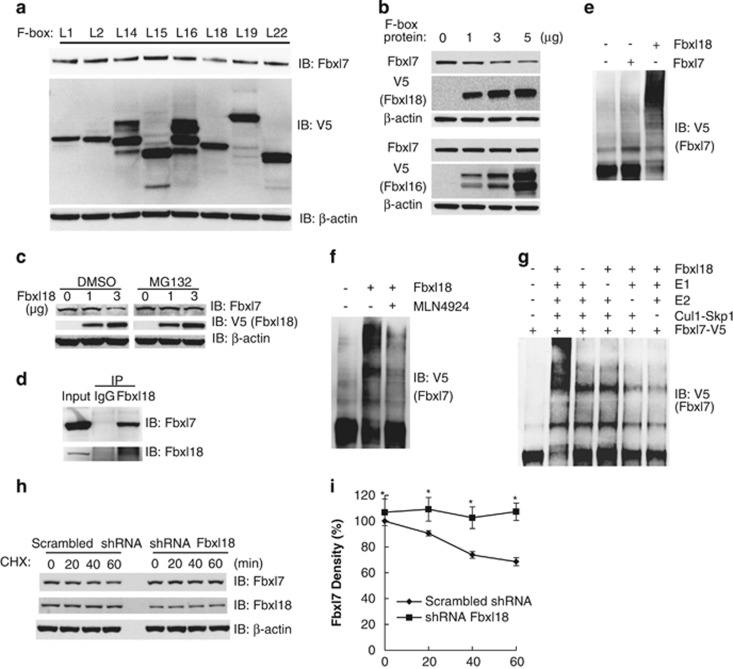
Fbxl18 targets Fbxl7 for polyubiquitylation and degradation.(**a**) MLE12 cells were transfected with V5-tagged *F-box* protein plasmids. Cell lysates were analyzed for Fbxl7, V5 and *β*-actin antibodies. (**b**) Cells were transfected with increasing amounts of *Fbxl18*-V5 or control *Fbxl16*-V5 plasmids. Twenty-four hours later, cell lysates were analyzed by V5, Fbxl7 and *β*-actin immunoblotting. (**c**) MLE12 cells were transfected with increasing amount of *Fbxl18*-V5 plasmids and incubated for 24 h. The cells were next treated with MG132 (20 *μ*M) for an additional 2 h before immunoblotting analysis by Fbxl7, V5 and *β*-actin antibodies. (**d**) Hela cell lysates were subjected to i.p. with endogenous Fbxl18 antibody and analyzed by Fbxl7 immunoblotting. (**e**) *In vitro* ubiquitylation assays. Each reaction contained ATP, ubiquitin, E1, E2 (UbcH5 and UbcH7), and Cul1-Rbx1, Skp1, non-tagged Fbxl7 or Fbxl18 as an E3 subunit, Fbxl7-V5 as a substrate. Polyubiquitylated Fbxl7 was detected by immunoblotting with V5 antibody. (**f**) Neddylation of Cul1 is required for polyubiquitylation of Fbxl7. Neddylation inhibitor MLN4924 (5 *μ*M) was used for non-neddylated Cul1 synthesis in a TNT reticulocyte lysate system. The polyubiquitylation of Fbxl7 with Cul1 or neddylation-deficient Cul1 (purified by Talon metal affinity resin) was detected by V5 immunoblotting. (**g**) *In vitro* ubiquitylation assays. Each control reaction lack the indicated component. (**h**,**i**) BEAS-2B cells were nucleofected with 4 *μ*g scrambled shRNA or shRNA Fbxl18#1 as indicated. Sixty hours later, Fbxl7 protein half-life was determined after Fbxl18 knockdown using shRNA, densitometry results of Fbxl7 immunoblots were plotted for half-life analysis in (**i**). Data are representative of two independent experiments. *P=0.042 (<0.05) *versus* scrambled shRNA

Recent studies indicate that some F-box proteins target others for their ubiquitylation and degradation.^12^ Thus, we investigated the interaction between Fbxl7 and randomly chosen F-box proteins by co-immunoprecipitation. Fbxl7 specifically bound to Fbxl18 (Supplementary Figure 1A). We further screened ability of ectopically expressed plasmids encoding ~50 F-box proteins from L, W and O families that might mediate Fbxl7 degradation. As shown in Figure 2a, randomly chosen V5-tagged F-box proteins in L family were ectopically expressed in MLE12 cells. Interestingly, only Fbxl18 overexpression decreased endogenous Fbxl7 protein levels. Additionally, we observed that overexpressed F-box proteins from W and O families displayed no effect on endogenous Fbxl7 protein abundance, as shown in Supplementary Figures 1B and C, respectively. We further observed that overexpression of *Fbxl18* plasmid decreased endogenous Fbxl7 protein levels in a dose-dependent manner (Figure 2b). However, transfection of cells with increasing amounts of *Fbxl16* plasmid had no effect on endogenous Fbxl7 levels. Particularly, MG132 treatment abolished the Fbxl7 decrease induced by overexpression of Fbxl18 (Figure 2c). In co-i.p. studies we detected the association of endogenous Fbxl18 with Fbxl7 (Figure 2d) in Hela cells. We further confirmed that Fbxl18 mediated the polyubiquitylation of Fbxl7 using *in vitro* ubiquitylation assays, and we also excluded the possibility that Fbxl7 might display autoubiquitylation activity (Figure 2e). Additionally, we investigated Fbxl18-mediated polyubiquitylation using a neddylation inhibitor MLN4924 (Figure 2f). Neddylation-deficient Cul1-Rbx1 largely decreased the polyubiquitylation of Fbxl7, in support that Fbxl7 polyubiquitylation is dependent on a fully functional SCF^Fbxl18^ complex. Fbxl7 polyubiquitylation was E1 and E2 dependent (Figure 2g). In separate studies knockdown of Fbxl18 with shRNA-stabilized Fbxl7 protein levels in a time-dependent manner (Figures 2h and i). These results indicate that Fbxl18 mediates Fbxl7 polyubiquitylation and proteasomal degradation.

### Lys^109^ is an Fbxl7 ubiquitin acceptor site

**Figure 3:**
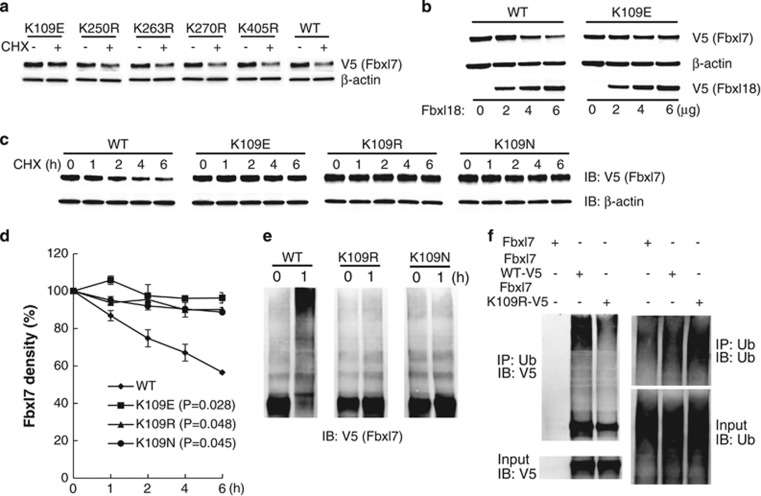
A ubiquitin acceptor site within Fbxl7 resides on the NH_2_-terminus of the F-box domain. (**a**) MLE12 cells were transfected with V5-tagged *Fbxl7* wild-type or *Fbxl7* single lysine mutant plasmids for 16 h, and then cells were treated with or without CHX (20 *μ*g/ml) for 6 h. Cell lysates were analyzed by V5 and *β*-actin antibodies using immunoblotting. (**b**) Fbxl7 protein levels were determined in cells after co-transfection with V5-tagged wild-type *Fbxl7* or *Fbxl7*^*K09E*^ plasmid with increasing amounts of *Fbxl18* plasmid. (**c**) Fbxl7 protein half-life was determined after expression of WT *Fbxl7*-V5, or *Fbxl7*^*K109*^-V5 mutant plasmids in cells and exposure to CHX. Each protein band on immunoblots was quantified densitometrically and shown graphically in (**d**) (data are representative of two independent experiments)*. P*<0.05 *versus* WT. (**e**) *In vitro* ubiquitylation assays. Each reaction contained ATP, ubiquitin, E1, E2, Cul1-Rbx1, Skp1 and non-tagged Fbxl18 with wild-type Fbxl7-V5, or Fbxl7^K109^-V5 mutant proteins as substrates (synthesized in TNT reticulocyte lysate and purified with Talon metal affinity resin). Polyubiquitylated Fbxl7 was analyzed by V5 immunoblotting. (**f**) *In vivo* ubiquitin pull down assay. MLE12 cells were transfected with non-tagged *Fbxl7*, *Fbxl7 WT-V5* and *Fbxl7 K109R-V5* plasmids. After 24 h incubation, the cells were treated with MG132 (20 *μ*M) for an additional 2 h before boiling in PBS containing 2% SDS. The lysates were next diluted in TBS to a final 0.2% SDS concentration before pull down using a rabbit ubiquitin antibody. The co-i.p. products were processed for immunoblotting with mouse V5 antibody and mouse ubiquitin antibodies, respectively

To localize the ubiquitin receptor site within Fbxl7, we ectopically co-expressed individual *Fbxl7* plasmids encoding lysine point mutations with an *Fbxl18* plasmid in MLE12 cells. Mutants whose protein levels decreased with overexpressed Fbxl18 were excluded from further analysis (data not shown). We then assessed protein stability of the remaining mutants by exposure to CHX treatment after *Fbxl7* cellular expression. We identified that only transfection of cells with a *Lys*^*109E*^ mutant stabilized the decay of Fbxl7, compared to the wild-type or other lysine mutants as shown in Figure 3a. Further, in MLE12 cells co-transfected with *Fbxl18* and *Fbxl7* plasmids, overexpressed *Fbxl18* plasmid dose-dependently decreased wild-type Fbxl7 levels, but had no effect on Fbxl7^K109E^ protein levels (Figure 3b). To exclude an amino acid charge effect on protein stability, we further compared the protein turnover rate after expression in cells with plasmids encoding additional Lys^109^ substitutions. As indicated in Figures 3c and d, expression in cells of plasmids encoding three distinct Lys^109^ point mutants resulted in stabilization of Fbxl7 proteins levels compared with cellular expression of wild-type *Fbxl7* plasmid. Here, the addition of a tag to the protein extended the lifespan of wild-type Fbxl7 *versus* endogenous Fbxl7 (Figure 1). In the *in vitro* ubiquitylation assays, the expressed Lys^109^ point mutants displayed resistance to SCF^Fbxl18^ –induced polyubiquitylation *versus* wild-type Fbxl7 (Figure 3e). We also compared the polyubiquitylation levels between wild-type Fbxl7 and the K109R mutant through ubiquitin pull downs in MLE12 cells. The lysine 109 mutation within Fbxl7 dramatically decreased Fbxl7 polyubiquitylation (Figure 3f). These results demonstrate that the ubiquitin acceptor site Lys^109^ is essential for Fbxl7 decay through ubiquitin-proteasomal processing.

### The FQ motif within Fbxl7 serves as the docking site for Fbxl18

**Figure 4:**
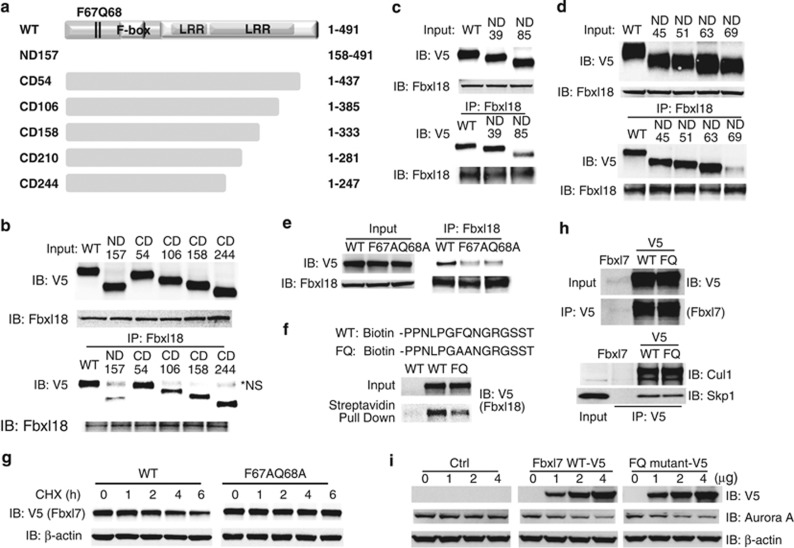
Fbxl18 binds an NH_2_-terminal FQ motif within Fbxl7.(**a**) Scheme represents truncated Fbxl7 mutants. (**b**)*-E*, V5-tagged truncated Fbxl7 or point mutation containing Fbxl7 was synthesized in reticulocyte lysate systems. Hela cell lysates were subjected to i.p. with Fbxl18 antibody and then mixed with *in vitro*-synthesized Fbxl7 mutants. Proteins were eluted with sample buffer and subjected to Fbxl18 immunoblotting for baits or V5 immunoblotting for truncated Fbxl7 (**b**, **c** and **d**) or FQ motif point mutants (**e**). (**f**) Peptides binding assays. Fbxl18-V5 (synthesized using TNT reticulocyte lysate and purified with Talon metal affinity resin) was mixed with biotin-labeled peptides (sequences as indicated) and pulled down by streptavidin resin. The eluted products were further processed for immunoblotting with V5 antibody. (**g**) Fbxl7 protein half-life was determined after expression of WT *Fbxl7*-V5, or *Fbxl7*^*F67AQ68A*^-V5 mutant plasmids. Data are representative of three independent experiments. (**h**) Fbxl7 FQ mutant preserves Cul1-Skp1 binding capacity. TNT reticulocyte lysate system synthesized non-tagged Fbxl7, Fbxl7 WT-V5 or Fbxl7 FQ-V5 proteins were mixed with MLE12 cell lysates and subjected to i.p. by V5 antibody. The co-i.p. products were immunoblotted by V5, Cul1, Skp1 antibodies respectively. (**i**) Both Fbxl7 WT and FQ mutant target Aurora A for degradation. Increasing amount of *pcDNA3.1* empty vector, *Fbxl7-V5*, *Fbxl7 FQ-V5* plasmids were transfected into MLE12 cells. Twenty-four hours later, the cell lysates were analyzed by immunoblotting with V5, Aurora A or *β*-actin antibody

Fbxl7 contains an NH_2_-terminal F-box domain and C-terminal LRRs. To identify the docking site of Fbxl18 on Fbxl7, we constructed a series of NH_2_-terminal and C-terminal truncated Fbxl7 expression plasmids, as schemed in Figure 4a. In Hela cells, endogenous Fbxl18 interacted with all the Fbxl7 C-terminal deletion mutants. However, loss of the NH_2_-terminal region containing the F-box domain largely abolished the interaction between Fbxl7 and Fbxl18 (Figure 4b). We then focused on the Fbxl7 NH_2_-terminal region and further localized the Fbxl18 interacting region within Fbxl7 via co-immunoprecipitation (Figures 4c and e). Figures 4d and e demonstrate that an amino-terminal region spanning residues 63–69 harbors a putative Fbxl18 binding site within Fbxl7. Indeed, by generating and expressing plasmids encoding specific Fbxl7 point mutants in cells followed by co.ip., we observed that an FQ motif within Fbxl7 was essential for Fbxl18 interaction with Fbxl7 (Figure 4e). To further confirm that the FQ motif is the interaction site between Fbxl18 and Fbxl7, we employed a biotin-labeled peptide binding assay and detected *in vitro* synthesized V5-tagged Fbxl18 bound to wild-type Fbxl7 peptides. However, a Fbxl7 FQ-AA mutant largely exhibited a decrease in its association with Fbxl18 (Figure 4f). We also compared Fbxl7 protein stability after expression in cells of a plasmid encoding either wild-type or an FQ mutant. As shown in Figure 4g, cellular expression of the FQ mutant plasmid resulted in an Fbxl7 variant that exhibited extended lifespan in cells *versus* the wild-type Fbxl7 protein. Moreover, similiar to wild-type Fbxl7, this FQ mutant displayed ability to retain association with other components within the SCF complex (Figure 4h) and to mediate degradation of a known substrate, Aurora A, for proteasomal degradation (Figure 4i). These results strongly suggest that an Fbxl7 FQ motif functions as the molecular recognition site for Fbxl18 binding. The binding capacity of Fbxl7 through this motif does not impact its effect on substrate degradation.

### Fbxl7 displays pro-apoptotic activity

**Figure 5:**
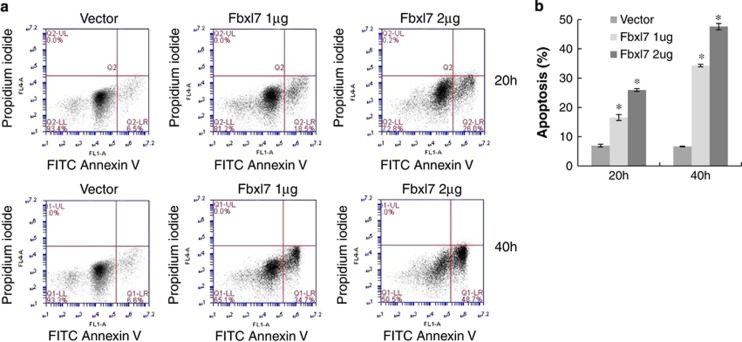
Ectopically expressed Fbxl7 induces apoptosis. (**a**,**b**) Hela cells were transfected with either 2-*μ*g control vector, 1- or 2 *-μ*g *Fbxl7*-V5 plasmid as indicated. After 20 h, 40 h incubation, cells were collected and stained with FITC-Annexin V and propidium iodine prior to analysis by flow cytometry. Data was quantitated and graphed (**b**). *P<0.001 *versus* vector

As we previously reported, ectopically expressed *Fbxl7* plasmid in cells causes mitotic arrest by targeting the mitotic spindle checkpoint kinase Aurora A for ubiquitylation and degradation.^19^ Here, we observed that overexpressed *Fbxl7* induces apoptosis. The expression of *Fbxl7* plasmid in cells led to appearance of a larger percentage of apoptotic cells in both a dose and time-dependent manner, as determined by propidium iodine staining compared with the cells transfected with a control vector (Figures 5a and b). These data indicate that Fbxl7 is pro-apoptotic.

### Fbxl18 counteracts Fbxl7-induced apoptosis

**Figure 6:**
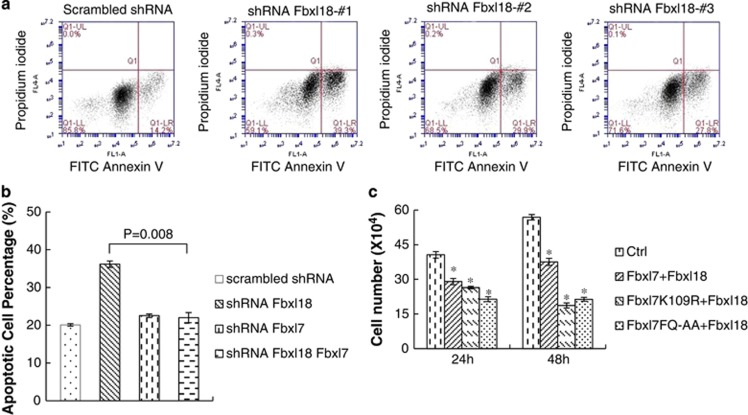
Fbxl18 counteracts the pro-apoptotic function of Fbxl7. (**a**) Hela cells were transfected with 3 *μ*g control scrambled shRNA or three shRNA targeting *Fbxl18* indicated as 1, 2 and 3. After 36 h, cells were collected and stained with FITC-Annexin V and propidium iodine for flow cytometry. (**b**) Hela cells were transfected with 3 *-μ*g control scrambled shRNA, or 3 *-μ*g shRNA targeting Fbxl7, Fbxl18, or both as indicated. 48 h later cells were collected and stained with FITC-Annexin V and propidium iodine for flow cytometry. Apoptotic cells percentage were quantified and plotted (*n=3*, *P<0.05 versus shRNA Fbxl18; data presented as mean±S.E.). (**c**) Proliferation studies of Hela cells after overexpression of *Fbxl7* wild-type or degradation-resistant mutant *Fbxl7* plasmids (dock site and acceptor site variants) and *Fbxl18* plasmid. Cells were collected, counted and stained with trypan blue. The viable cells were counted and quantified. (*n*=4; *P<0.05 *versus* control (Ctrl); data presented as mean±S.E.)

As shown above, overexpression of *Fbxl7* plasmid causes apoptosis. We next investigated if Fbxl18 differentially modulates Fbxl7-induced apoptosis. Cellular depletion of Fbxl18 with three different Fbxl18 shRNAs caused increased apoptosis, compared with effects of scrambled shRNA-treated cells (Figure 6a). However, the apoptosis induced by knockdown Fbxl18 was largely rescued by the additional knockdown of Fbxl7 (Figure 6b). Further, as shown in Figure 6c, double transfection of the *Fbxl7 FQ* mutant and *Fbxl18* plasmids showed a impaired cellular proliferation, compared with the cells transfected with *Fbxl7* wild-type and *Fbxl18* plasmids or a control vector. These data suggest that the Fbxl7 docking site mutant protein that lacks ability to interact with Fbxl18 harbors an extended lifespan in cells to induce apoptosis. Further, Fbxl18 counteracts the pro-apoptotic activity of Fbxl7 in a biological model of cell injury.

## Discussion

Fbxl7 is a highly evolutionally conserved protein sharing a 98% sequence identity between mouse and humans, and >95% identity among other species. However, Fbxl7 is a relatively uncharacterized protein with regard to its biological role and molecular regulation in cells. Whether Fbxl7 is an oncoprotein or a component within a complex that triggers mitotic arrest leading to cell death requires further study. Our data demonstrate potent activity of this SCF component as it is sufficient to elicit apoptosis in some cells. This effect is relevant as high-level Fbxl7 protein expression in cells, left unchecked, could promote sustained tissue injury through activation of apoptotic programs. This is important given that cell proliferative activity must overcome the activities of pro-apoptotic factors to ensure optimal healing during tissue injury. Here, we demonstrate that Fbxl7 protein levels are controlled by the actions of the SCF^Fbxl18^ complex, perhaps serving as a feedback control mechanism to limit high-level expression of this pro-apoptotic factor. We have demonstrated that Fbxl7 harbors a molecular signature that recruits Fbxl18, identified the acceptor site for Fbxl18 activity within its substrate, and present data suggesting that levels of Fbxl18 affect the ability of cells to remain viable from Fbxl7-induced apoptosis. The data are also the first to characterize the molecular behavior of Fbxl18 in cells and raise the possibility for molecular targeting of an Fbxl18***—*****|**Fbxl7→apoptosis pathway.

Fbxl7 is a relatively short-lived protein (t^1/2^~1 h) through SCF^Fbxl18^-dependent polyubiquitylation and subsequent proteasomal degradation. However, 1 h after cycloheximide treatment, Fbxl7 protein levels remains stable in cells (Figures 1a and b). One possibility could be that Fbxl7 interacts with ubiquitin-specific peptidases. For example, Fbxl7 directly associates to USP1 and USP45, and indirectly is linked to USP12.^22^ These deubiquitylation enzymes could counteract SCF^Fbxl18^ to remove ubiquitin from Fbxl7 to prevent its degradation. More importantly, the Fbxl7 steady-state levels remain detectable with some degree of stabilization following its initial rapid decay that appears to be caused by the accelerated degradation of Fbxl18 based on our observations (data not shown). Although the half-life of endogenous Fbxl7 is short, the addition of a C-terminal V5 tag extended its half-life to ~6 h. This might result from a conformation change induced by the tag or that exogenous Fbxl7 exceeded the endogenous SCF^Fbxl18^ ubiquitylation machinery capacity to impair its function. The underlying mechanism for this disparity is under further investigation.

The molecular factors that regulate Fbxl18 levels and potential sensing of Fbxl7 abundance also remain obscure. Fbxl18 may be prone to autoubiquitylation or other modifications and subsequent degradation. Our data suggest that Fbxl18 levels rapidly fluctuate in cells but when elevated, they are linked to stability of Fbxl7 to regulate the apoptotic program. We speculate that there may exist a negative feedback loop driven by Fbxl18 that could be essential to keep a dynamic equilibrium of Fbxl7, which could be critical for execution of vital roles by multiple substrates downstream Fbxl7. The identification of these Fbxl7 substrates in addition to Aurora A also requires further study.

The interaction of F-box protein and its substrates must be tightly controlled. In response to a stimuli, F-box proteins must recruit substrates rapidly and specifically to the catalytic core for ubiquitylation and degradation. For example, phosphorylation of the substrate resulting in a phosphodegron is one of the most common recognition signals for F-box proteins, which either accelerates or inhibits the interaction of F-box protein and substrates. In response to a specific stimuli, kinases such as glycogen synthase kinase 3*β* regulate biological function by manipulating protein stability through phosphorylating substrates.^23, 24^ Recently, acetylation is also considered as an important post-translational modification to regulate protein stability.^25^ Additionally, other interaction factors also have a vital role in mediating F-box protein to recognize their substrates.^26, 27^ Interestingly, another F-box protein, Fbxl2 that impacts lipogenesis, innate immune responses and cell cycle progression targets its substrates through an IQ motif on its substrates.^28^ In this study, we identified that a similar FQ motif within Fbxl7 is the major docking site for recognition by Fbxl18. We cannot completely exclude the possibility that another minor domain within Fbxl7 might also serve as a recognition signal to recruit Fbxl18 because a series of N-terminal deletions do not totally eliminate the interaction between Fbxl18 and Fbxl7 (Figures 4b–f), and C-terminal truncated Fbxl7 mutants showed slightly different binding intensities to Fbxl18. Whether this FQ motif-based interaction mechanism is unique or undergoes any modification responding to stimuli, is currently under further investigation. The results suggest that some members of the F-box family harboring LRR domains may be recruited to their substrates through these signatures.

The ability of Fbxl18 to recruit pro-apoptotic Fbxl7 for ubiquitylation and proteasomal degradation may be of fundamental importance in human disease. For example, aside from neoplasia, several disorders including emphysema, traumatic injury and aging are characterized by an imbalance between cell proliferative activities and apoptosis. The ability of Fbxl18 to protect cells from apoptosis induced by Fbxl7 might provide an opportunity to design small molecule Fbxl18 agonists to enhance tissue repair and regeneration, or alternatively design novel therapeutics that impair Fbxl18:Fbxl7 interaction to stimulate apoptosis in tumors. These data are, however, too preliminary to conclude that Fbxl18 is an anti-apoptotic molecule until larger numbers of Fbxl18-substrate pairs are characterized in multiple systems. We are currently investigating the identities of additional SCF^Fbxl18^ substrates to better interpret its role in apoptosis. Additionally, the Fbxl7 sequence is highly conserved among species. Specifically, Fbxl7 in mammals is homologous to Grr1 in yeast *Saccharomyces cerevisiae*. Grr1 is among the best-studied F-box proteins in yeast, which has a vital role in cell cycle regulation by recruiting Cln2.^29^ Besides Aurora A, we expect to identify additional kinases or cell cycle regulators under regulation of Fbxl7 to further elucidate the pro-apoptotic behavior of Fbxl7.

## Materials and Methods

### Cells

Murine lung epithelial (MLE12) cells (ATCC) and human bronchial epithelial cells (BEAS-2B) were cultured with HITES medium containing 10% fetal bovine serum (FBS) and antibiotics. Hela cells were cultured with Eagle's Minimum Essential Medium (EMEM) medium containing 10% FBS and antibiotics.

### Reagents

V5 antibody, the pcDNA3.1D/V5-His-TOPO cloning kit, and *E. coli* Top10 One-Shot competent cells were from Invitrogen (St. Louis, MO, USA). HA mouse antibody, ubiquitin, Cul1, Skp1, and Aurora A antibodies, stress and apoptosis signaling antibody array kits were from Cell Signaling (Danvers, MA, USA). Leupeptin, HA rabbit antibody and *β*-actin mouse monoclonal antibody were from Sigma (Carlsbad, CA, USA). MG-132 was from UBPBio (Aurora, CO, USA). The F-box proteins cDNA, scrambled shRNA and human Fbxl18 shRNAs were from OpenBiosystems (Huntsville, AL, USA). QuickChange site-directed mutagenesis kits were from Agilent (Santa Clara, CA). The FuGENE HD Transfection Reagent and TNT Quick Coupled Transcription/Translation Systems were from Promega (Madison, WI, USA). FITC-Annexin V Apoptosis Detection Kit with PI was from Biolegend (San Diego, CA, USA). Immobilized protein A/G beads were from Pierce (Rockford, IL, USA). Tris pH 7.6, MgCl_2_, DTT, ATP, ubiquitin-activating enzyme, UbcH5, UbcH7, ubiquitin, and ubiquitin aldehyde were from Enzo Life Sciences (Farmingdale, NY, USA). Skp1, Cul1, Rbx1 were from Abnova (Taipei, Taiwan). Talon metal affinity resin was from Clontech (Mountain View, CA, USA). Cycloheximide was from Calbiochem (La Jolla, CA, USA). Fbxl7 mouse monoclonal antibody, *β*-TrCP, Fbxo3, Fbxo15, Fbxo28 antibodies and control IgG were from Santa Cruz Biotechnology (Santa Cruz, CA, USA). Fbxl7 rabbit polyclonal antibody was from Novus (Littleton, CO, USA). Fbxl18 rabbit polyclonal antibody was from Abcam (Cambridge, MA, USA). Fbxl14 antibody was from Proteintech (Chicago, IL, USA). Fbxo7 rabbit polyclonal antibody was from Aviva Systems Biology (San Diego, CA, USA).

### Cloning and mutagenesis

PCR-based approaches were applied to clone different F-box proteins into pcDNA3.1D/v5-his (Invitrogen) for expression in mammalian cells using appropriate primers. All mutants were constructed using PCR-based approaches or site-directed mutagenesis (Agilent).

### shRNAs

Scrambled shRNA: TRC lentiviral human shRNA. TRC Human FBXL18 shRNA#1 (Clone ID: TRCN0000155770; mature antisense: AAACTGAAGTAGAACGGGTTG). TRC Human FBXL18 shRNA#2 (Clone ID: TRCN0000156053; mature antisense: ACGTTCAGAATCAGATCTGTG). TRC Human FBXL18 shRNA#3 (Clone ID: TRCN0000156441; mature antisense: TGCAGTGCTTCAACATGTCTG).

### Transfection

All plasmids were delivered into MLE12 cells or BEAS-2B cells using nucleofection or FuGENE HD (Hela cells), following the manufacturers' protocols. Cellular expression of GFP-tagged plasmids using these methods was achieved at >90% efficiency.

### *In vitro* ubiquitylation assay

The assays were performed in a volume of 20 *μ*l containing 50 mM Tris pH 7.6, 5 mM MgCl_2_, 0.6 mM DTT, 2 mM ATP, 400 *μ*M MG-132, 50 nM ubiquitin-activating enzyme, 0.5 *μ*M UbcH5, 0.5 *μ*M UbcH7, 2 *μ*M ubiquitin, and 1 *μ*M ubiquitin aldehyde, 20 nM Cul1, 20 nM Rbx1, 20 nM Skp1, and *in vitro* synthesized FBXL7-V5, and Fbxl18 within the TNT coupled reticulocyte system. TNT-synthesized proteins were purified via Talon metal affinity resin and reaction products were processed for V5 immunoblotting.

### Immunoblotting and Immunoprecipitation

Cell lysates (normalized to total protein concentration) were subjected to SDS-PAGE, electrotransferred to membranes, and immunoblotted. For ubiquitin pull down, the freshly collected cells in PBS containing 2% SDS were boiled at 100 °C for 10 min. The lysates were diluted with TBS to a final concentration with 0.2% SDS containing 5 *μ*M ubiquitin aldehyde. 1 mg total cellular proteins were immunoprecipitated with 2 *μ*g ubiquitin rabbit antibodies at 4 °C for 3–4 h followed by the addition of 30 *μ*l of protein A/G-agarose for an additional 1 h at 4 °C. The precipitated complex was washed three times with 0.5% Triton X-100 in PBS and analyzed by immunoblotting with an enhanced ECL system. For other immunoprecipitations, 1-mg cell lysates in PBS containing 0.5% Triton X-100 were incubated with 2*-μ*g-specific primary antibodies for 3–4 h at 4 °C followed by the addition of protein A/G-agarose and three washes with lysis buffer before immunoblotting.

### Apoptosis analysis

Transfected cells were incubated with FITC-Annexin V and propidium iodide for 15 min following manufacturer's protocols (Biolegend, San Diego, CA, USA). FACS samples were analyzed with the AccuriC6 system with *De Novo* Software. For proliferation assays, Hela cells were transfected with indicated plasmids. Cells were cultured in 35 mm dishes for up to 48 h; at indicated time points, cells were collected and stained with trypan blue. Viable cells were then counted and quantified.

### Statistical analysis

Statistical comparisons were performed using an ANOVA or an unpaired *t*-test with *P*<0.05 indicative of significance.

## Abbreviations

ANOVA: analysis of variance
LRR: leucine-rich repeat
CHX: cycloheximide
co-i.p.: co-immunoprecipitation
EMEM: Eagle's Minimum Essential Medium
FBS: fetal bovine serum
Fbxo: F-box only
MLE: murine lung epithelial
SCF: Skp-Cullin- F box
Ub: ubiquitin
WD: tryptophan-aspartic acid, WT, wild type

## References

[bib1] 1Tanaka Y, Tanaka N, Saeki Y, Tanaka K, Murakami M, Hirano T et al. c-Cbl-dependent monoubiquitination and lysosomal degradation of gp130. Mol Cell Biol 2008; 28: 4805–4818. 18519587 10.1128/MCB.01784-07 PMC2493370

[bib2] 2Hershko A, Ciechanover A, Varshavsky A. Basic Medical Research Award. The ubiquitin system. Nat Med 2000; 6: 1073–1081. 11017125 10.1038/80384

[bib3] 3Hochstrasser M. Biochemistry. All in the ubiquitin family. Science 2000; 289: 563–564. 10939967 10.1126/science.289.5479.563

[bib4] 4Tyers M, Willems AR. One ring to rule a superfamily of E3 ubiquitin ligases. Science 1999; 284: 603–604. 10.1126/science.284.5414.601 10328744

[bib5] 5Deshaies RJ, Joazeiro CA. RING domain E3 ubiquitin ligases. Annu Rev Biochem 2009; 78: 399–434. 19489725 10.1146/annurev.biochem.78.101807.093809

[bib6] 6Cardozo T, Pagano M. The SCF ubiquitin ligase: insights into a molecular machine. Nat Rev Mol Cell Biol 2004; 5: 739–751. 15340381 10.1038/nrm1471

[bib7] 7Zheng N, Schulman BA, Song L, Miller JJ, Jeffrey PD, Wang P et al. Structure of the Cul1-Rbx1-Skp1-F boxSkp2 SCF ubiquitin ligase complex. Nature 2002; 416: 703–709. 11961546 10.1038/416703a

[bib8] 8Skowyra D, Craig KL, Tyers M, Elledge SJ, Harper W. F-box proteins function as receptors to recruit phosphorylated substrates to E3 ubiquitin ligase complexes. Mol Biol Cell 1997; 8: 2059–2059. 10.1016/s0092-8674(00)80403-1 9346238

[bib9] 9Wang Z, Liu P, Inuzuka H, Wei W. Roles of F-box proteins in cancer. Nat Rev Cancer 2014; 14: 233–247. 24658274 10.1038/nrc3700 PMC4306233

[bib10] 10Weathington NM, Mallampalli RK. Emerging therapies targeting the ubiquitin proteasome system in cancer. J Clin Invest 2014; 124: 6–12. 24382383 10.1172/JCI71602 PMC3871250

[bib11] 11Lipkowitz S, Weissman AM. RINGs of good and evil: RING finger ubiquitin ligases at the crossroads of tumour suppression and oncogenesis. Nat Rev Cancer 2011; 11: 629–643. 21863050 10.1038/nrc3120 PMC3542975

[bib12] 12Chen BB, Coon TA, Glasser JR, McVerry BJ, Zhao J, Zhao Y et al. A combinatorial F box protein directed pathway controls TRAF adaptor stability to regulate inflammation. Nat Immunol 2013; 14: 470–479. 23542741 10.1038/ni.2565 PMC3631463

[bib13] 13Cenciarelli C, Chiaur DS, Guardavaccaro D, Parks W, Vidal M, Pagano M. Identification of a family of human F-box proteins. Curr Biol 1999; 9: 1177–1179. 10531035 10.1016/S0960-9822(00)80020-2

[bib14] 14Ilyin GP, Rialland M, Glaise D, Guguen-Guillouzo C. Identification of a novel Skp2-like mammalian protein containing F-box and leucine-rich repeats. FEBS Lett 1999; 459: 75–79. 10508920 10.1016/s0014-5793(99)01211-9

[bib15] 15Yen HC, Xu Q, Chou DM, Zhao Z, Elledge SJ. Global protein stability profiling in mammalian cells. Science 2008; 322: 918–923. 18988847 10.1126/science.1160489

[bib16] 16Skaar JR, Pagan JK, Pagano M. Mechanisms and function of substrate recruitment by F-box proteins. Nat Rev Mol Cell Biol 2013; 14: 369–381. 23657496 10.1038/nrm3582 PMC3827686

[bib17] 17Bashir T, Dorrello NV, Amador V, Guardavaccaro D, Pagano M. Control of the SCF(Skp2-Cks1) ubiquitin ligase by the APC/C(Cdh1) ubiquitin ligase. Nature 2004; 428: 190–193. 15014502 10.1038/nature02330

[bib18] 18Galan JM, Peter M. Ubiquitin-dependent degradation of multiple F-box proteins by an autocatalytic mechanism. Proc Natl Acad Sci USA 1999; 96: 9124–9129. 10430906 10.1073/pnas.96.16.9124 PMC17743

[bib19] 19Coon TA, Glasser JR, Mallampalli RK, Chen BB. Novel E3 ligase component FBXL7 ubiquitinates and degrades Aurora A, causing mitotic arrest. Cell Cycle 2012; 11: 721–729. 22306998 10.4161/cc.11.4.19171 PMC3318106

[bib20] 20Kim S, Kon M, DeLisi C. Pathway-based classification of cancer subtypes. Biol Direct 2012; 7: 21. 22759382 10.1186/1745-6150-7-21 PMC3485163

[bib21] 21Park HW, Dahlin A, Tse S, Duan QL, Schuemann B, Martinez FD et al. Genetic predictors associated with improvement of asthma symptoms in response to inhaled corticosteroids. J Allergy Clin Immunol 2014; 133: 664–669 e665. 24486069 10.1016/j.jaci.2013.12.1042 PMC4112383

[bib22] 22Tan MK, Lim HJ, Bennett EJ, Shi Y, Harper JW. Parallel SCF adaptor capture proteomics reveals a role for SCFFBXL17 in NRF2 activation via BACH1 repressor turnover. Mol Cell 2013; 52: 9–24. 24035498 10.1016/j.molcel.2013.08.018 PMC3981468

[bib23] 23Zhao J, Wei J, Mialki RK, Mallampalli DF, Chen BB, Coon T et al. F-box protein FBXL19-mediated ubiquitination and degradation of the receptor for IL-33 limits pulmonary inflammation. Nat Immunol 2012; 13: 651–658. 22660580 10.1038/ni.2341 PMC3643313

[bib24] 24Busino L, Millman SE, Scotto L, Kyratsous CA, Basrur V, O'Connor O et al. Fbxw7alpha- and GSK3-mediated degradation of p100 is a pro-survival mechanism in multiple myeloma. Nat Cell Biol 2012; 14: 375–385. 22388891 10.1038/ncb2463 PMC3339029

[bib25] 25Zou C, Chen Y, Smith RM, Snavely C, Li J, Coon TA et al. SCF(Fbxw15) mediates histone acetyltransferase binding to origin recognition complex (HBO1) ubiquitin-proteasomal degradation to regulate cell proliferation. J Biol Chem 2013; 288: 6306–6316. 23319590 10.1074/jbc.M112.426882 PMC3585065

[bib26] 26Ganoth D. The cell-cycle regulatory protein Cks1 is required for SCFSkp2-mediated ubiquitinylation of p27. (vol 3, pg 321, 2001). Nat Cell Biol 2001; 3: 438–438. 10.1038/35060126 11231585

[bib27] 27Spruck C, Strohmaier H, Watson M, Smith AP, Ryan A, Krek TW et al. A CDK-independent function of mammalian Cks1: targeting of SCF(Skp2) to the CDK inhibitor p27Kip1. Mol Cell 2001; 7: 639–650. 11463388 10.1016/s1097-2765(01)00210-6

[bib28] 28Chen BB, Coon TA, Glasser JR, Mallampalli RK. Calmodulin antagonizes a calcium-activated SCF ubiquitin E3 ligase subunit, FBXL2, to regulate surfactant homeostasis. Mol Cell Biol 2011; 31: 1905–1920. 21343341 10.1128/MCB.00723-10 PMC3133224

[bib29] 29Kishi T, Yamao F. An essential function of Grr1 for the degradation of Cln2 is to act as a binding core that links Cln2 to Skp1. J Cell Sci 1998; 111: 3655–3661. 9819356 10.1242/jcs.111.24.3655

