# Supplementary figures and images for: F-box protein Fbxl18 mediates polyubiquitylation and proteasomal degradation of the pro-apoptotic SCF subunit Fbxl7
Source: Cell Death Dis. 2015 Feb 5;6(2):e1630–. doi: 10.1038/cddis.2014.585 (PMC4669792; doi:10.1038/cddis.2014.585)

Supplementary Figure 1

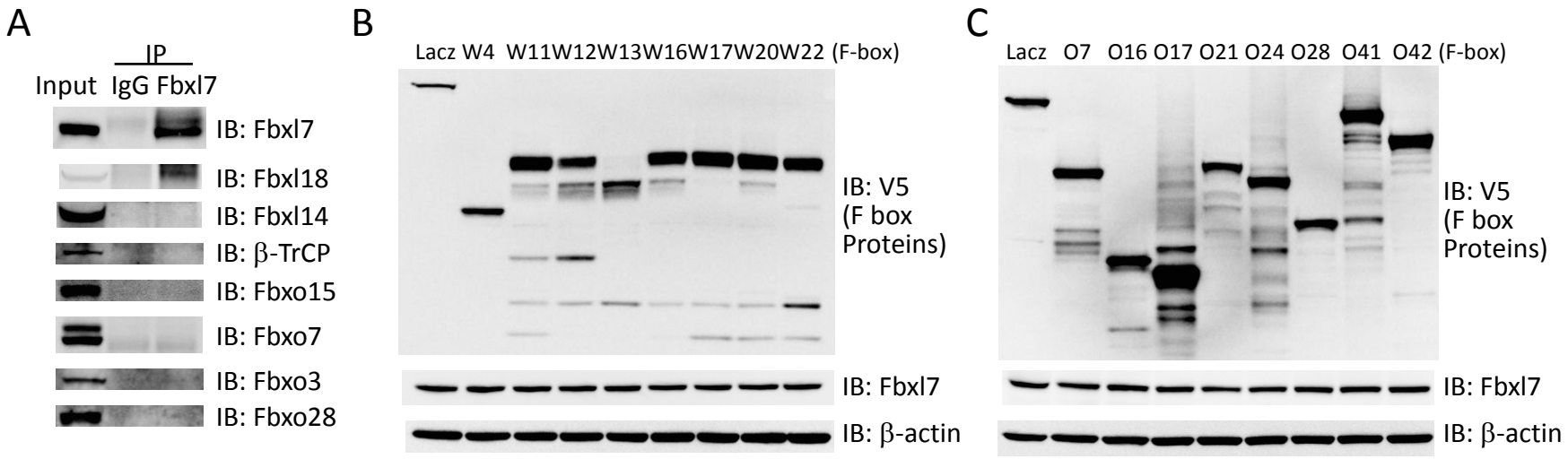

Supplement: Supplementary Figure 1 [file cddis2014585x1.pdf]
