# Supplementary material for: F-box protein Fbxl18 mediates polyubiquitylation and proteasomal degradation of the pro-apoptotic SCF subunit Fbxl7
Source: Cell Death Dis. 2015 Feb 5;6(2):e1630–. doi: 10.1038/cddis.2014.585 (PMC4669792; doi:10.1038/cddis.2014.585)
Supplement: Supplementary Figure Legend [file cddis2014585x2.docx]

**Supplementary Figure 1**. Fbxl18 specifically associates with Fbxl7 and regulates its protein abundance.

*A,* Hela cell lysates were subjected to co-i.p. with either mouse IgG control or mouse Fbxl7 antibody. The immunoprecipitated products and input lysates were processed for immunoblotting with indicated F-box antibodies. *B-C,* MLE12 cells were transfected with V5 tagged W or O family F box protein plasmids. After 16 h incubation, the cell lysates were determined by V5, Fbxl7, or β-actin immunoblotting.
